# Feasibility and Acceptance of Self-Guided Mobile Ultrasound among Pregnant Women in Routine Prenatal Care

**DOI:** 10.3390/jcm12134224

**Published:** 2023-06-22

**Authors:** Constanza A. Pontones, Adriana Titzmann, Hanna Huebner, Nina Danzberger, Matthias Ruebner, Lothar Häberle, Bjoern M. Eskofier, Michael Nissen, Sven Kehl, Florian Faschingbauer, Matthias W. Beckmann, Peter A. Fasching, Michael O. Schneider

**Affiliations:** 1 Department of Obstetrics and Gynaecology, Universitätsklinikum Erlangen, 91054 Erlangen, Germany; adriana.titzmann@uk-erlangen.de (A.T.); hanna.huebner@uk-erlangen.de (H.H.); nina.danzberger@uk-erlangen.de (N.D.); matthias.ruebner@uk-erlangen.de (M.R.); lothar.haeberle@uk-erlangen.de (L.H.); sven.kehl@uk-erlangen.de (S.K.); florian.faschingbauer@uk-erlangen.de (F.F.); matthias.beckmann@uk-erlangen.de (M.W.B.); peter.fasching@uk-erlangen.de (P.A.F.); michael.schneider@uk-erlangen.de (M.O.S.); 2 Machine Learning and Data Analytics Lab, Friedrich-Alexander-Universität Erlangen-Nürnberg, 91052 Erlangen, Germany; bjoern.eskofier@fau.de (B.M.E.); michael.nissen@fau.de (M.N.)

**Keywords:** mobile ultrasound, self-guided ultrasound, pregnancy, prenatal care, feasibility, acceptance

## Abstract

Background and objectives: Mobile and remote ultrasound devices are becoming increasingly available. The benefits and possible risks of self-guided ultrasound examinations conducted by pregnant women at home have not yet been well explored. This study investigated aspects of feasibility and acceptance, as well as the success rates of such examinations. Methods: In this prospective, single-center, interventional study, forty-six women with singleton pregnancies between 17 + 0 and 29 + 6 weeks of gestation were included in two cohorts, using two different mobile ultrasound systems. The participants examined the fetal heartbeat, fetal profile and amniotic fluid. Aspects of feasibility and acceptance were evaluated using a questionnaire. Success rates in relation to image and video quality were evaluated by healthcare professionals. Results: Two thirds of the women were able to imagine performing the self-guided examination at home, but 87.0% would prefer live support by a professional. Concerns about their own safety and that of the child were expressed by 23.9% of the women. Success rates for locating the target structure were 52.2% for videos of the fetal heartbeat, 52.2% for videos of the amniotic fluid in all four quadrants and 17.9% for videos of the fetal profile. Conclusion: These results show wide acceptance of self-examination using mobile systems for fetal ultrasonography during pregnancy. Image quality was adequate for assessing the amniotic fluid and fetal heartbeat in most participants. Further studies are needed to determine whether ultrasound self-examinations can be implemented in prenatal care and how this would affect the fetomaternal outcome

## 1. Introduction

Ultrasound is used in routine prenatal care to monitor fetal growth and to detect fetal abnormalities and other pathologies during pregnancy [1]. Ultrasound examinations are carried out by qualified professionals since the handling of the devices and interpretation of the images are complex and require training and experience [2].

Technology continues to advance worldwide and is becoming more important in the healthcare sector. Mobile and remote medical devices are currently being introduced in prenatal medicine [3,4]. The COVID-19 pandemic might have accelerated this development as alternative designs for prenatal care in accordance with social distancing were needed [5,6]. Various mobile ultrasound devices have been developed and are being used in several countries (mainly for nonmedical self-scanning) as they are becoming more affordable. With an option for remote guidance by professionals, there would also be an opportunity to obtain imaging that might be usable for clinical care [7]. This seems to offer potential advantages, such as enhanced access to healthcare, especially in rural areas, with subsequent reduced visits to outpatient clinics and fewer hospital admissions, which might lead to cost savings in the healthcare system [3].

Although initial research results have shown that mobile health devices are acceptable and feasible for use as home monitoring tools to improve prenatal care [8,9], potential risks for pregnant women and for unborn children should not be disregarded [10].

### Purpose

The aim of this study was to investigate the feasibility and acceptance, as well as success rates, of medically supervised self-examinations with mobile ultrasound devices during pregnancy, with the participating women being provided with detailed instructions in advance.

## 2. Methods

### 2.1. Study Design and Population

This was an open, prospective, single-center, interventional cohort study. Women with singleton pregnancies aged from 18 to 50 years and in gestational weeks 17 + 0 to 29 + 6 weeks were included. Twin pregnancies were ineligible. The participants were recruited from two settings: women who were hospitalized due to pregnancy-related complications and women presenting for planned antenatal care. The methods were performed in accordance with the relevant guidelines and regulations and approved by the ethics committee of Friedrich-Alexander-Universität Erlangen-Nürnberg (ref. Number 299_20 B). The participants were enrolled into two cohorts, each using one of two different mobile ultrasound devices (cohort A: Instinct, PulseNmore Ltd., Omer, Israel; cohort B: Butterfly iQ, Butterfly Network Inc., Guilford, CT, USA). A total of 47 pregnant women were screened and 46 were enrolled (23 in each cohort), as one patient did not wish to participate in the study.

### 2.2. Study Procedures and Data Collection

After undergoing a routine ultrasound examination by an experienced examiner assessing the fetal heart rate, biometry, echocardiography, fetal profile and amniotic fluid, pregnant women matching the inclusion criteria were asked to participate in the study. They were given information about the study and written informed consent was obtained. An ultrasound device, a mobile phone and an ultrasound gel were handed to the participant. They received direct instruction on how to use the device and the following tasks were explained over 5–10 min using example images:

- Recording a video of the fetal heartbeat for 30 s;
- Taking a picture and recording a video of the fetal profile for 60 s;
- Taking a picture and recording a 15-s video of the amniotic fluid in each of the four quadrants of the abdomen.

There was only one examination that was completed at the study visit on site. The examination was stopped after 15 min, regardless of whether all of the tasks had been completed. The participants were asked to complete a questionnaire about age, number of pregnancies and parity, demographic background, educational level and experience in using electronic devices. Questions about the feasibility and acceptance of the self-guided ultrasound examination were also included. The study ended with the submission of the questionnaire.

The images and videos obtained were saved on the study phone and then transferred to the study server. The videos were saved as MP4 files and the images were saved as PNG or JPG files. After successful storage, the original recordings were deleted from the study smartphone. The patient data collected were pseudonymized and documented in a database.

### 2.3. Ultrasound Devices and Settings

Cohort A: Instinct (PulseNmore Ltd.) is a mobile ultrasound system that was developed for home use. It consists of a mobile ultrasound device that can be connected to a mobile phone or tablet (only Android devices are currently applicable). The corresponding app can be downloaded free of charge. The device did not have any preinstalled presets for ultrasound in obstetrics and an individual study setting was therefore created and used uniformly for all participants according to the manufacturer’s recommendations: gain 80%, frequency 3 MHz, power 0 dB, focus 50–90 mm, depth 150 mm, frame averaging off, line density 1, rejection 5, dynamic range 60 dB, image enhancement 7, time gain compensation 50% (all), gamma 1.16, speckle reduction 4, contrast 0, map 3, brightness 20%.

Cohort B: Butterfly iQ (Butterfly Network, Inc., Manufacturer: Butterfly Network, city: Burlington, MA, USA) is a mobile ultrasound system that is certified for medical use. It consists of a mobile ultrasound device that can be connected to a mobile phone or tablet by cable (compatible with both Apple and Android devices). The corresponding app can be downloaded free of charge. The cloud function for saving images and videos can be deactivated. The preset for ultrasound in obstetrics was used (frequency 1.7/3.4 MHz, 240 acquired receive lines per frame, dynamic range 32 to 48 dB).

### 2.4. Evaluation of Image and Video Quality

**Figure 1:**
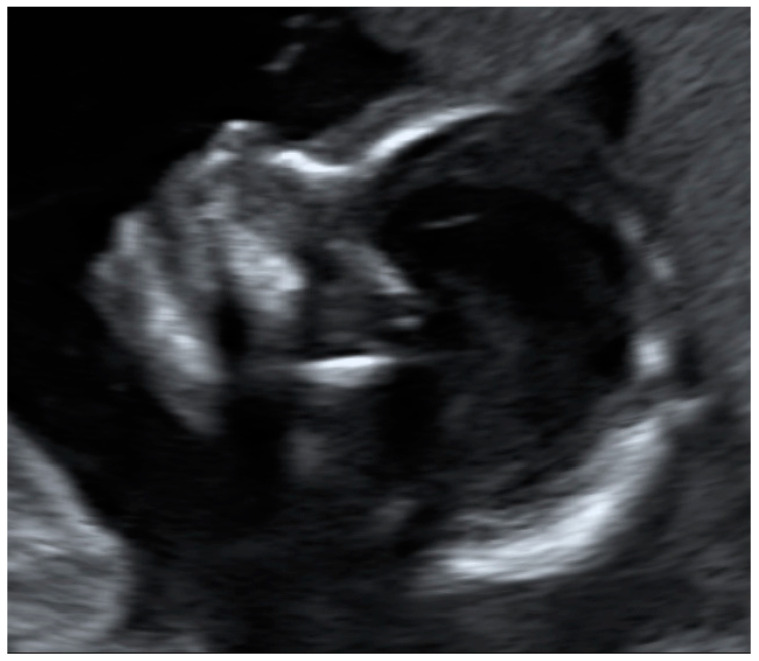
Requirements for satisfactory presentation of fetal profile: the forehead, tip of the nose and chin should be clearly visible.

All images and videos obtained during the self-examination were evaluated by one experienced ultrasound examiner after study recruitment had been completed. Each image or video was assessed individually and was marked as follows:

- Images or videos that showed the required target structure (e.g., heartbeat, fetal profile or amniotic fluid) were marked as “target located.”
- Images or videos in which the required target structure was not shown were marked as “target structure not located.”
- Images or videos that showed the required target structure, but with poor quality that was not suitable for medical assessment, were marked as “target structure located, but quality low” (e.g., the image quality was blurred, or the target structure was not completely visible).
- If the participant was able to show the fetal heartbeat for at least one second during the video sequence lasting 30 s, it was marked as “target located.” For a satisfactory presentation of the fetal profile, the forehead, tip of the nose and chin had to be clearly visible (example in Figure 1). Images and videos taken by the participants in which the fetal profile was also not visible (*n* = 18) were excluded.
- For a satisfactory presentation of images and videos of the amniotic fluid, hypoechoic areas had to be clearly demarcated from parts of the fetus. The four images and four video sequences representing the amniotic fluid were evaluated separately. If the amniotic fluid was clearly displayed in one quadrant, the respective videos or image sequences were marked as “target located.” It was also examined whether the women were able to display the amniotic fluid in all four quadrants so that retrospective evaluation of a normal amount of amniotic fluid in a participant would be possible. Accordingly, the videos were marked as “4 out of 4 with sufficient quality” only if the amniotic fluid was visible in all four video sequences.

### 2.5. Data Analysis

A descriptive statistical analysis of the maternal characteristics and answers given in the questionnaire was carried out. Descriptive statistics (mean, standard deviation, frequency, percentages) were calculated. The success rate in relation to image and video quality in the examinations performed by the pregnant women was also reported using descriptive statistics.

## 3. Results

All participants completed the required study procedures (self-guided ultrasound examination and questionnaire). They all managed to complete the tasks assigned in ≤ 15 min. No device-related serious adverse events were noted during the examination.

### 3.1. General Maternal Characteristics

The participants’ characteristics are shown in Table 1. The mean maternal age was 32.6 ± 5.2 years. The mean gestational age was 24.0 ± 3.2 weeks. For more than one third of the patients, it was their first (39.1%) or second pregnancy (34.8%). With regard to educational level, nearly half of the participants (41.3%) had an academic degree and 8.7% had a doctoral degree.

### 3.2. Evaluation of the Questionnaire on Feasibility and Acceptance

The questionnaire responses are shown in Table 2. Nearly half of the women in cohort B (43.5%) and nearly one third of those in cohort A felt confident using the ultrasound device. A total of 7 of the 46 women (15.2%) felt uncertain when performing the examination. In all, 26.1% of the participants in cohort A and 17.4% of those in cohort B said they would only be willing to carry out the examination with supervision by a physician. Two thirds of the participants (67.4%) could imagine carrying out the self-guided examination at home. A total of 87.0% would like the attending physician to provide live support via video telephony if they were performing the examination at home.

The participants were asked whether it would be acceptable for them not to see the images before the evaluation of the scans by a physician, which could take a few hours or days (e.g., using the offline mode five-step scanning procedure). A total of 34.8% said that this would not be acceptable.

With regard to patients’ opinions about safety, 33 of the 46 participants (71.7%) did not think that the examinations might be harmful to them or to the unborn child in any way, while 23.9% were slightly concerned about their own safety and that of the child.

### 3.3. Assessment of Image and Video Quality

**Figure 2:**
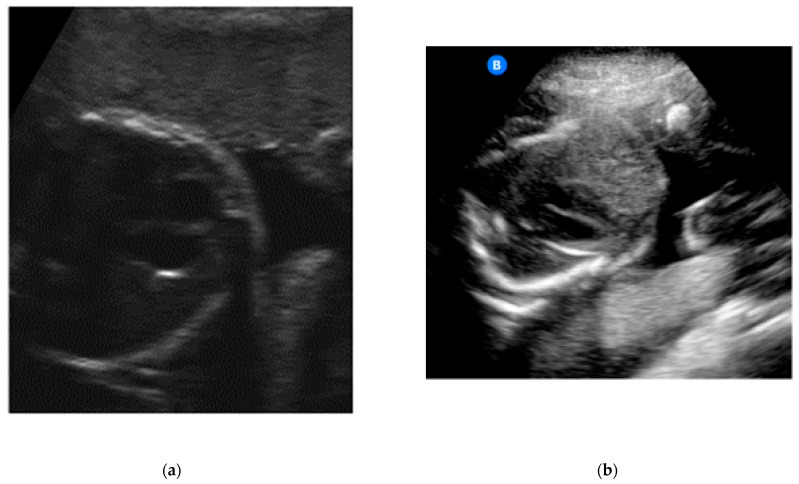
(**a**) Example of an image obtained by a study participant in cohort A, showing the heartbeat (satisfactory presentation). (**b**) Example of an image obtained by a study participant in cohort B, showing the heartbeat (satisfactory presentation).

The heartbeat was correctly captured by more than half of the participants (*n* = 24, 52.2%; Table 3) (Figure 2a,b).

**Figure 3:**
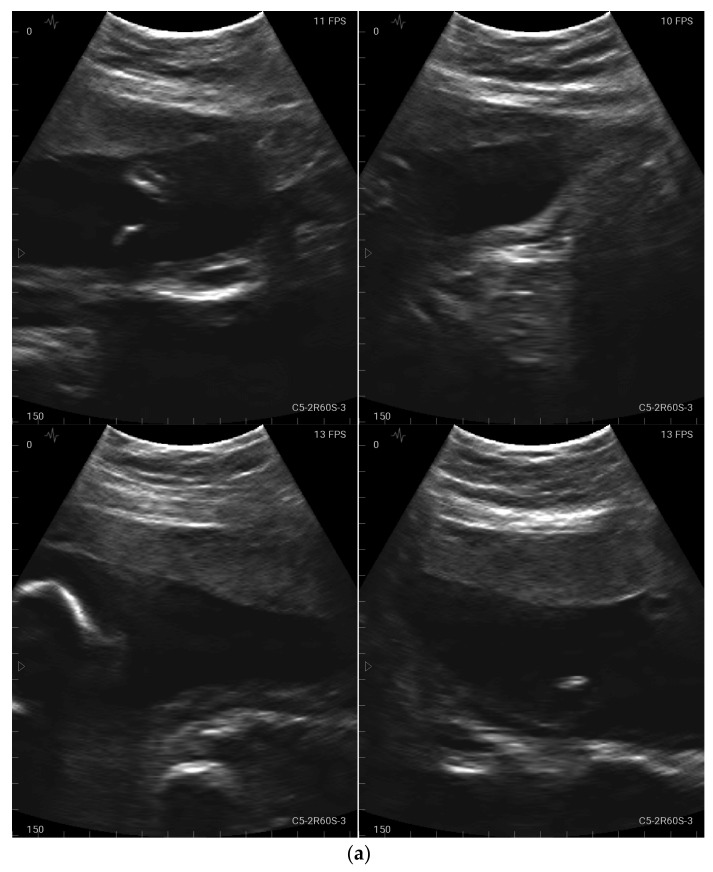

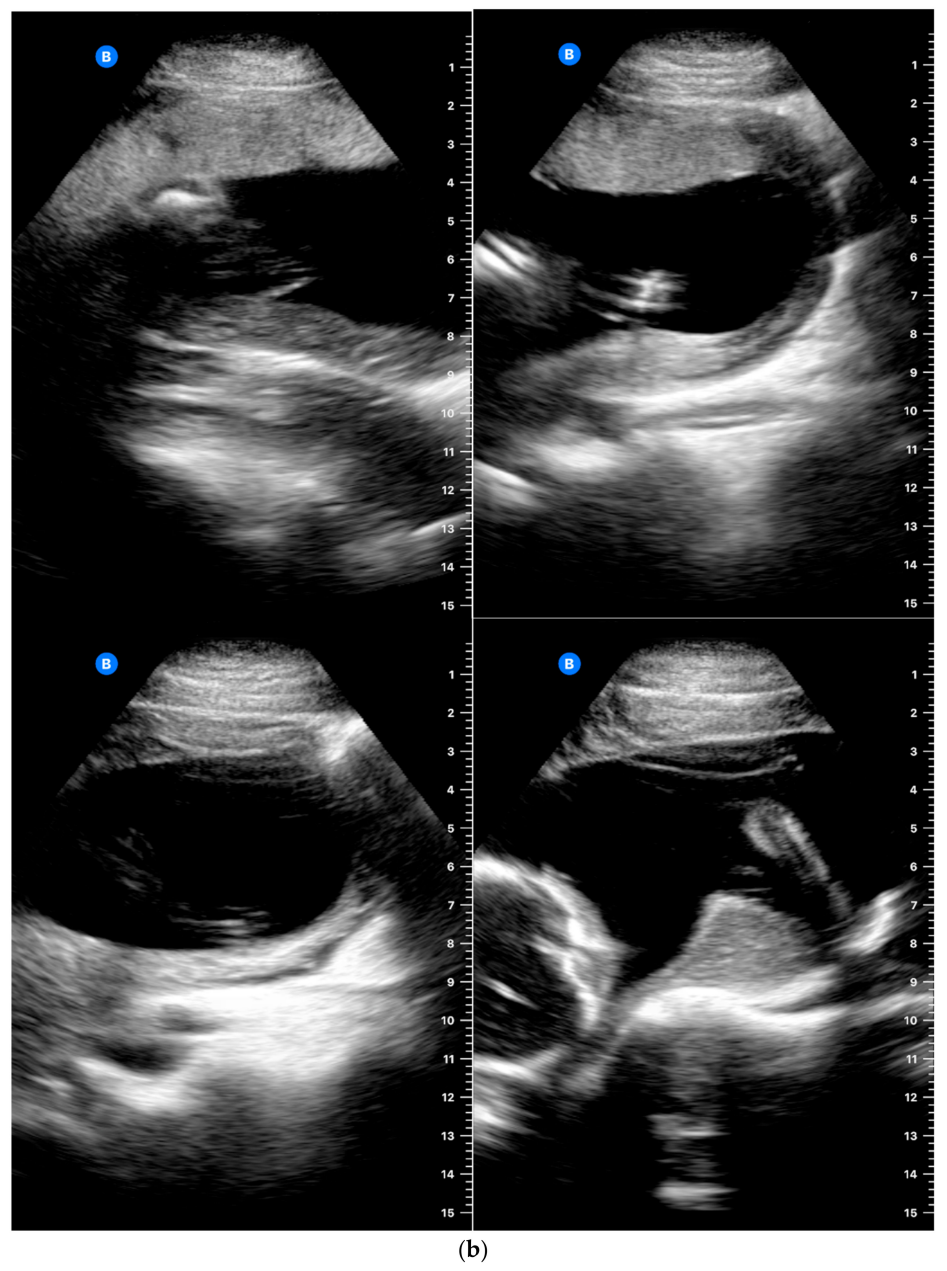
(**a**) Example of an image obtained by a study participant in cohort A, showing the amniotic fluid (satisfactory presentation). (**b**) Example of an image obtained by a study participant in cohort B, showing the amniotic fluid (satisfactory presentation).

With regard to identifying the amniotic fluid during the 15-s video, 43 of the 46 participants (93.5%) managed to locate the amniotic fluid correctly in at least one of the four quadrants (Figure 3a,b). A total of 52.2% succeeded in locating the amniotic fluid in all four quadrants in the videos. In relation to capturing still images of the amniotic fluid, the rate of images with sufficient quality was lower than with the videos (80.4% with at least one adequate still image in the four quadrants and 43.5% with sufficient quality in all four quadrants).

**Figure 4:**
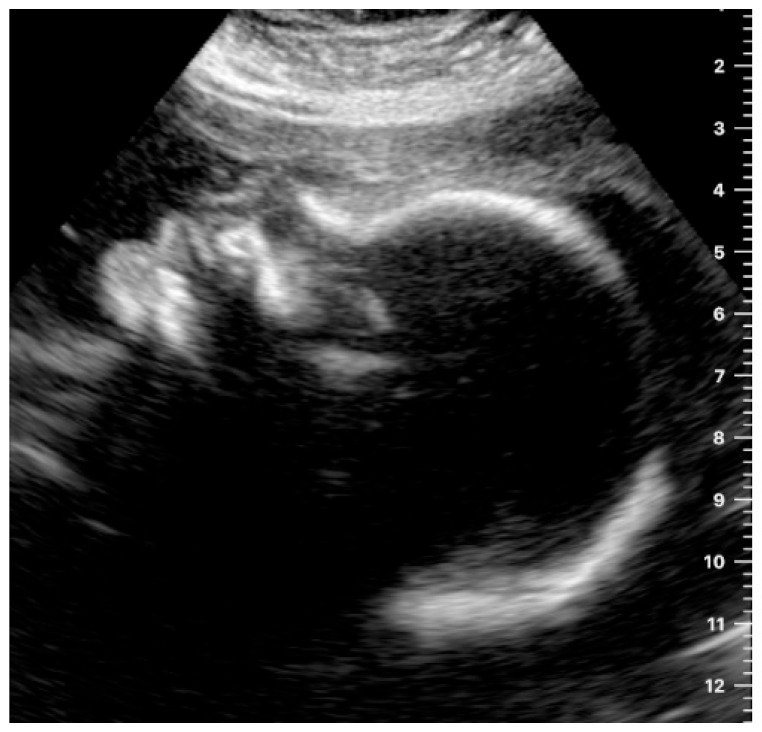
Example of an image obtained by a study participant, showing the fetal profile (satisfactory presentation).

The fetal profile was located satisfactorily by 14.3% of the women in still images (an example is shown in Figure 4) and by 17.9% in the videos.

## 4. Discussion

In this study of the feasibility and acceptability of self-guided mobile ultrasound during pregnancy, two thirds of the pregnant women would be willing to do the self-examination alone at home; however, the majority would prefer the attending physician to provide live support. Nearly half of the women felt confident using the ultrasound device and few women had concerns about their own safety or that of the child. The success rates for locating the target structure were better for the fetal heartbeat and the amniotic fluid than for the fetal profile.

The finding that the majority of the pregnant women (87.0%) would prefer live support during the self-examination is in line with a 2019 survey by Schramm et al. including 509 women, in which skeptical attitudes toward pregnancy self-monitoring were reduced when the procedure was combined with web-based consultation with a physician [11].

With regard to the accuracy of self-examination with ultrasound devices in the present study, the success rate for locating the amniotic fluid (43.5% for images, 52.2% for videos) and fetal heartbeat (52.2% for videos) was good as this was the first time handling the ultrasound device and even trainees in obstetrics and gynecology need more than 24 months of clinical experience to manage ultrasound examinations independently [12]. In particular, displaying the fetal profile is known to be technically demanding [13] and this was accordingly the most challenging task with low success rates (14.3% for images, 17.9% for videos). Depending on factors such as the fetal position, even experienced sonographers cannot always visualize the fetal face sufficiently (frequency of incomplete visualization 6–9% [14,15]).

A similar clinical trial conducted in Israel in 2020–2021 using the Instinct ultrasound system included 100 women with a singleton pregnancy at 14 + 0 to 39 + 6 gestational weeks [16]. In that study, the success rate for detection was 95.3% for fetal heart activity (much higher than in our results in the Instinct cohort, at 26.1%), 88.3% for body movements, 69.4% for tone, 92.2% for normal amniotic fluid volume (similar to our results) and 23.8% for breathing movements. The self-assessed user experience was rated at 4.4/5, whereas device satisfaction was rated at 3.9/5 [16]. As in the present study, each participant received personal face-to-face instruction on how to use the device at the time of recruitment. The first ultrasound scan was performed with guidance from an experienced ultrasound examiner, in contrast to the present study. The women were allowed to perform several scans at home (with a minimum of one and a maximum of three per day, limited to 3 min per scan) over a period of 7–14 days during pregnancy. Before each scan, the women viewed animated video demonstrations of how to move the device across the maternal abdomen. The mean number of scans per participant was 13.6 ± 6.2 each. There were no device-related serious adverse events [16]. There were two additional differences between the study by Hadar et al. and this one: Firstly, the women were allowed to use the mobile device at home up to three times per day, which might influence the stress level and pressure to succeed in performing the self-examination. Secondly, being able to use the device several days in a row might affect the women’s learning curve in comparison with a single attempt to perform the examination. It can be assumed that it is much easier to learn how to locate the amniotic fluid than how to detect the fetal heartbeat.

The use of mobile devices in medicine has already been reported to be acceptable and feasible in other investigations, particularly in high-risk groups and specific patient groups. One example is the use of self-operated endovaginal telemonitoring during fertility treatments. In a small pilot study including 15 women, a good correlation was observed between the number of follicles measured in a self-operated ultrasound and in an ultrasound performed by a professional. The procedure also appeared to be more patient-friendly and less time-consuming [17].

Areas in which mobile ultrasound devices may be useful in the prenatal context include situations that require close fetal monitoring. Cuneo et al. investigated the use of mobile devices in pregnant women who have antibodies associated with the risk of a fetal atrioventricular block developing [18]. In the study, which included 315 pregnant women with positive anti-Ro antibodies, fetal heart rate and rhythm were measured twice a day using a portable Doppler device. A total of 87% of the patients completed the monitoring protocol, which did not increase their anxiety levels. Abnormal fetal heart rates and rhythms were detected by 6.7% of the women. No cases of atrioventricular block were missed during home monitoring. Many of the patients managed to reach a hospital within less than 12 h after a fetal atrioventricular block occurred [18]. This example shows that self-monitoring may be able to reduce the number of clinical consultations and even identify fetal pathologies during pregnancy earlier.

Further potential risks during home surveillance of fetal parameters using mobile devices also need to be taken into account. Prospective randomized trials are needed in order to analyze whether self-guided examinations with mobile ultrasound devices reach at least the same levels of sensitivity and specificity as standard-of-care examinations by professionals in the healthcare system. In a retrospective analysis in which 105 cases of fetal gastroschisis were accompanied by daily home monitoring of the fetal heart rate, the false-positive rate of fetal distress at admission was reported to be 58%. A significant increase in the rate of cesarean sections was also observed (50% vs. 24%), without any influence on perinatal outcome parameters such as the Apgar score or umbilical artery pH at birth [19].

Future studies should also investigate whether ultrasound self-monitoring influences anxiety among pregnant women, either positively or negatively. Several studies showed that increased stress and anxiety in pregnancy have been associated with poor birth outcomes and restricted fetal growth [20]. Prenatal anxiety might also have an impact on the number of emergency consultations on the one hand and on postnatal depression on the other. In a survey from 2019, women were asked if they would visit the emergency department less often if smart devices were readily available and only 7.7% affirmed [11]. With regard to depression, it is known that antenatal anxiety is related to postnatal depression [21,22]. Xu et al. found that women who presented to the emergency room during pregnancy were more likely to be admitted to hospital for a diagnosis of postnatal depression [23].

Finally, adverse events due to increased exposure to ultrasonic waves themselves also need to be taken into account. Diagnostic levels of ultrasound can lead to increased temperatures in tissue and nonthermal effects of ultrasound have also been demonstrated in animals. Although no hazardous effects have been demonstrated in humans to date, the European Federation of Societies for Ultrasound in Medicine and Biology (EFSUMB) stated in 2020 that little information is available regarding possible subtle biological effects of ultrasound on the developing human embryo or fetus, so that the exposure time should be limited as much as possible [24].

### Strengths and Limitations

Although the study population was small in each cohort in the present study, each of the participants managed to complete all the tasks so that the images and videos could be fully evaluated. The educational level was high, as half of the participants had an academic degree. This may have had a positive influence on the success rate of performing ultrasound self-examinations. The scans were also performed immediately after having a presentation by trained staff—this may also impact the success rate and should be taken into consideration in future studies.

## 5. Conclusions

Self-examination using mobile systems for fetal ultrasound during pregnancy was generally acceptable for the pregnant women who participated. The analysis showed that the image quality was adequate for assessing amniotic fluid in most participants. Identification of fetal heartbeat and fetal facial profile was more challenging for the women. Further studies are needed to determine whether ultrasound self-examinations can be implemented in prenatal care and what effects this might have on fetomaternal outcomes.

The next step will be to perform a clinical trial using study procedures similar to those in this study, but with the women receiving additional live support from a physician using video telephony.

## Figures and Tables

**Table 1:** Maternal characteristics in 46 participants.

Characteristic	Cohort A and B (*n* = 46)		Cohort A (*n* = 23)		Cohort B (*n* = 23)	
	Mean or *n* ^1^	SD or % ^1^	Mean or *n* ^1^	SD or % ^1^	Mean or *n* ^1^	SD or % ^1^
Maternal age	32.6	5.2	33.8	5.0	31.4	5.1
Gestational week	24.0	3.2	24.1	3.3	23.9	3.0
Pregnancy						
1	18	39.1	8	34.8	10	43.5
2	16	34.8	8	34.8	8	34.8
≥3	12	26.1	7	30.4	5	21.7
Educational level						
No school-leaving qualification	0	0	0	0	0	0
Lower secondary school qualification	1	2.2	0	0	1	4.3
Intermediate school qualification	6	13.0	3	13.0	3	13.0
University entrance qualification	4	8.7	3	13.0	1	4.3
Apprenticeship qualification	12	26.1	5	21.7	7	30.4
Bachelor’s/master’s degree	19	41.3	10	43.5	9	39.1
Doctoral degree	4	8.7	2	8.7	2	8.7
Smartphone ownership						
No smartphone	0	0	0	0	0	0
iOS	20	43.5	8	34.8	12	52.2
Android	23	50.0	14	60.9	9	39.1
iOS and Android	2	4.3	1	4.3	1	4.3
Unknown	1	2.2	0	0	1	4.3

^1^ Mean and standard deviation (SD) are shown for continuous characteristics and frequency (*n*) and percentage (%) for categorical characteristics.

**Table 2:** Feasibility and acceptability of self-guided ultrasound among pregnant women.

Questions	Cohort A and B (*n* = 46)		Cohort A (*n* = 23)		Cohort B (*n* = 23)	
	*n*	%	*n*	%	*n*	%
How confident did you feel using the ultrasound probe?						
Very confident	2	4.3	2	8.7	0	0
Confident	17	37.0	7	30.4	10	43.5
Partly/partially	20	43.5	8	34.8	12	52.2
Unsure	7	15.2	6	26.1	1	4.3
Very unsure	0	0	0	0	0	0
Could you imagine doing this examination at home by yourself?						
Yes	31	67.4	14	60.9	17	73.9
No	10	21.7	5	21.7	5	21.7
I do not know	5	10.9	4	17.4	1	4.3
If you were to perform this examination at home, would you like the attending physician to provide live support via video telephony?						
Yes	40	87.0	20	87.0	20	87.0
No	2	4.3	1	4.3	1	4.3
I do not know	4	8.7	2	8.7	2	8.7
If you were doing this examination at home, would it be okay for you if the doctor first had to evaluate and approve the ultrasound image before you could see it?						
Yes	21	45.7	9	39.1	12	52.2
No	16	34.8	9	39.1	7	30.4
I do not know	9	19.6	5	21.7	4	17.4
Do you agree with the following statements?						
The self-examination was fun						
I completely agree	31	67.4	14	60.9	17	73.9
Agree	12	26.1	7	30.4	5	21.7
Neither agree nor disagree	3	6.5	2	8.7	1	4.3
A little	0	0	0	0	0	0
Not at all	0	0	0	0	0	0
I would like to do the self-examination more often						
I completely agree	18	39.1	8	34.8	10	43.5
Agree	15	32.6	8	34.8	7	30.4
Neither agree nor disagree	5	10.9	3	13.0	2	8.7
A little	8	17.4	4	17.4	4	17.4
Not at all	0	0	0	0	0	0
The self-examination took too much time						
I completely agree	1	2.2	0	0	1	4.3
Agree	2	4.3	1	4.3	1	4.3
Neither agree nor disagree	1	2.2	0	0	1	4.3
A little	15	32.6	10	43.5	5	21.7
Not at all	27	58.7	12	52.2	15	65.2
I was afraid of doing something wrong during the self-examination						
I completely agree	1	2.2	1	4.3	0	0
Agree	3	6.5	2	8.7	1	4.3
Neither agree nor disagree	10	21.7	4	17.4	6	26.1
A little	13	28.3	5	21.7	8	34.8
Not at all	19	41.3	11	47.8	8	34.8
I would only do the self-examination under the supervision of a doctor						
I completely agree	10	21.7	6	26.1	4	17.4
Agree	8	17.4	5	21.7	3	13.0
Neither agree nor disagree	12	26.1	5	21.7	7	30.4
A little	5	10.9	3	13.0	2	8.7
Not at all	11	23.9	4	17.4	7	30.4
I am concerned that the self-examination may be harmful to me or the child						
I completely agree	0	0	0	0	0	0
Agree	0	0	0	0	0	0
Neither agree nor disagree	2	4.3	2	8.7	0	0
A little	11	23.9	4	17.4	7	30.4
Not at all	33	71.7	17	73.9	16	69.6
I would like to do the self-examination at home						
I completely agree	18	39.1	7	30.4	11	47.8
Agree	9	19.6	6	26.1	3	13.0
Neither agree nor disagree	11	23.9	6	26.1	5	21.7
A little	7	15.2	4	17.4	3	13.0
Not at all	1	2.2	0	0	1	2.3

**Table 3:** Evaluation of image and video quality.

Evaluation	Cohort A and B (*n* = 46)		Cohort A (*n* = 23)		Cohort B (*n* = 23)	
	*n*	%	*n*	%	*n*	%
Amniotic fluid—images						
Target structure located	37	80.4	17	73.9	20	87.0
Target structure not located	3	6.5	1	4.3	2	8.7
Target structure located, but quality low	6	13.0	5	21.7	1	4.3
Amniotic fluid—videos						
Target structure located	43	93.5	20	87.0	23	100
Target structure not located	1	2.2	1	4.3	0	0
Target structure located, but quality low	2	4.3	2	8.7	0	0
Amniotic fluid—total images						
4 out of 4 with sufficient quality	20	43.5	8	34.8	12	52.2
Amniotic fluid—total videos						
4 out of 4 with sufficient quality	24	52.2	10	43.5	14	60.9
Heartbeat—videos						
Target structure located	24	52.2	6	26.1	18	78.3
Target structure not located	10	21.7	8	34.8	2	8.7
Target structure located, but quality low	12	26.1	9	39.1	3	13.0
Fetal profile—images	28		13		15	
Target structure located	4	14.3	1	7.7	3	20.0
Target structure not located	23	82.1	11	84.6	12	80.0
Target structure located, but quality low	1	3.6	1	7.7	0	0
Fetal profile—videos	28		13		15	
Target structure located	5	17.9	2	15.4	3	20.0
Target structure not located	23	82.1	11	84.6	12	80.0
Target structure located, but quality low	0	0	0	0	0	0

## Data Availability

The datasets generated during and/or analyzed during this study are available from the corresponding author upon reasonable request.
